# High volume nasal irrigations with steroids for chronic rhinosinusitis and allergic rhinitis

**DOI:** 10.1007/s00405-024-08901-9

**Published:** 2024-09-26

**Authors:** Giacomo Bertazzoni, Carlo Conti, Gabriele Testa, Giorgia Carlotta Pipolo, Davide Mattavelli, Cesare Piazza, Luca Pianta

**Affiliations:** 1 Department of Otorhinolaryngology, Azienda Socio Sanitaria Territoriale di Cremona, Cremona, Italy; 2 Department of Medical and Surgical Specialties, Radiological Sciences, and Public Health, Università degli Studi di Brescia, Brescia, Italy; 3 Unit of Otorhinolaryngology, Head and Neck Surgery, Azienda Socio Sanitaria Territoriale Spedali Civili Di Brescia, Brescia, Italy; 4 Unit of Otorhinolaryngology, Head and Neck Surgery, Azienda Socio Sanitaria Territoriale Santi Paolo e Carlo, Milan, Italy; 5 Department of Heath Sciences, Università Statale degli Studi di Milano, Milan, Italy

**Keywords:** Chronic rhinosinusitis, Nasal irrigations, Allergic rhinitis, Nasal corticosteroids

## Abstract

**Purpose:**

The primary aim of this systematic review is to assess the efficacy, safety, and cost-effectiveness of high-volume steroid nasal irrigation (SNI) for treating chronic rhinosinusitis (CRS) and allergic rhinitis (AR).

**Methods:**

A systematic review of literature from 2012 to 2024 was conducted using PubMed to identify relevant studies. The search focused on terms related to AR, CRS, and steroid nasal irrigation. Studies were screened for relevance and duplicates removed, resulting in 20 studies being included in the final analysis. These studies were categorized based on their focus on efficacy, safety, or both, and underwent a risk of bias assessment using Cochrane and ROBINS-I tools.

**Results:**

Of the 20 studies included, 13 examined the effectiveness of high-volume nasal steroid irrigations, 4 investigated safety, and 3 covered both. High-volume irrigations demonstrated superior efficacy in symptom improvement for CRS and AR compared to nasal sprays, particularly post-surgery. Budesonide was the most commonly used steroid. Safety evaluations indicated no significant hypothalamic–pituitary–adrenal axis suppression or increases in intraocular pressure, although minor adverse events were reported.

**Conclusion:**

High-volume steroid nasal irrigations are more effective than standard nasal sprays for CRS and AR, particularly post-surgery, without significant safety concerns. However, no studies on cost-effectiveness were found, suggesting a need for further research in this area.

## Introduction

First-line medical treatment for chronic rhinosinusitis and allergic rhinitis aims at symptom control either as an alternative to surgery or as post-operative maintenance therapy. Standard therapy typically includes intranasal corticosteroids administered with nasal spray devices, which are effective in reducing inflammation and improving symptoms [1].

Nasal irrigation is an alternative topical therapy delivery technique, designed to deliver with high pressure a high-volume solution of saline and corticosteroid (HVSC) into the nasal cavities. This method, usually performed with 250 ml squeeze bottles, is thought to offer several potential benefits over nasal sprays, including increased drug delivery, especially in the post-sinus surgery setting [2]. While both delivery methods have shown efficacy in managing nasal symptoms, the comparative effectiveness and safety of these treatments have been scarcely investigated [3–5].

The primary aim of this systematic review is to assess current evidence regarding efficacy, safety and cost-effectiveness of steroid nasal irrigation (SNI) in the treatment not only of chronic rhinosinusitis (CRS) [6] but also in allergic rhinitis (AR).

## Materials and methods

**Fig. 1:**
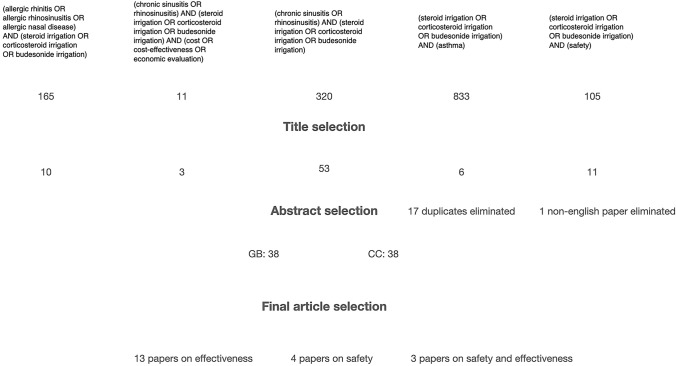
Article selection process

A systematic review was conducted using a search strategy aimed at identifying studies evaluating the efficacy, safety profiles and cost-effectiveness of corticosteroid nasal irrigations used for chronic rhinosinusitis and allergic rhinitis published from 2012 to 2024 in English. On January 15th, 2024, a search of available literature was carried out using the PubMed search engine by two of the investigators (GB and CC). Five different searches were conducted. The search strings were: “(allergic rhinitis OR allergic rhinosinusitis OR allergic nasal disease) AND (steroid irrigation OR corticosteroid irrigation OR budesonide irrigation)”, “(chronic sinusitis OR rhinosinusitis) AND (steroid irrigation OR corticosteroid irrigation OR budesonide irrigation) AND (cost OR cost-effectiveness OR economic evaluation)”, “(chronic sinusitis OR rhinosinusitis) AND (steroid irrigation OR corticosteroid irrigation OR budesonide irrigation)”, “(steroid irrigation OR corticosteroid irrigation OR budesonide irrigation) AND (asthma)”, “(steroid irrigation OR corticosteroid irrigation OR budesonide irrigation) AND (safety)”. All retrieved studies were first screened to remove any duplicate records. The first screening of the literature was done by excluding titles that were evidently not pertinent to the review objectives. Using the same criteria, a second screening was done by reading the abstracts of the remaining articles and a third screening was performed by reading selected articles. After the three screenings the two investigators compared and discussed the final articles’ selection (Fig. 1) by including in the final analysis only randomized clinical studies and cohort studies. The details of each of the retained studies were reported using 3 structured templates, one for reports on SNI efficacy in CRS (Table 1), one for efficacy in AR (Table 2) and the third concerning SNI overall safety (Table 3). A risk of bias (RoB) assessment was done for every study included in the final analysis. Three different RoB tools were employed: Revised Cochrane risk-of-bias tool for randomized trials (RoB 2) for randomized clinical trials, a Cochrane tool to assess risk of bias in cohort studies and the Risk of Bias in Non-randomized Studies–of Interventions (ROBINS-I) assessment tool. Each article was then classified as having “high”, “unclear” or “low” risk of bias (Table 4).

**Table 1:** Steroid high-volume irrigations in chronic rhino sinusitis–efficacy

Study	Year	Study type	Patient selection criteria	Patients number	Mean age (years)	Type of steroid	Daily dose	Delivery method	Concomitant systemic steroid use	Duration of study (weeks)	Comparison	Primary endpoint	Main results
Snidvongs et al. [7]	2012	Case series (prospective)	Patients with CRSwNP and CRSsNP undergoing ESS after failing medical therapy	111	50.1 ± 13.5	Budesonide or Betamethasone	Budesonide 1 mg or Betamethasone 1 mg	240 ml squeeze bottle	None	55.5 ± 33.9	Pretreatment state	Symptom improvement (questionnaire, SNOT22)	Significant symptom improvement
Jang et al. [8]	2013	Case series (retrospective)	Patients with CRSwNP and CRSsNP after ESS	60	45 (17–73)	Budesonide	1 mg	90 ml lavage with syringe	Variable	108	Same patients during treatment lapses > than 1 month	SNOT20 and Lund Kennedy endoscopic score (LKES)	Overall benefit on symptom score during Budesonide irrigation use
Tait et al. [9]	2018	Randomized Clinical Trial	Patients with CRSwNP and CRSsNP without ESS in the prior 6 weeks and antibiotics in the prior 2 weeks	79	51 ± 14.7	Budesonide + saline vs placebo + saline	1 mg	240 ml squeeze bottle	None	4	With placebo arm and pretreatment state	Intraparticipant change of SNOT22 compared between budesonide and control groups	Clinically meaningful improvement in self- reported functional status and quality of life measures as well as objective measurements of CRS
Zi‐zhen Huang et al. [10]	2018	Case series (prospective)	Patients with CRSwNP and CRSsNP after ESS	60	Experimental group: 37.15 ± 8.623; Control group 38.10 ± 15.579	Budesonide + saline vs saline alone	Not reported	Not reported	Variable	12	Comparison between experimental and control group	Post-op Lund-Kennedy endoscopico score, VAS for nasal blockage, hyposmia and rhinorrhea, SNOT22, SF-36, SAS. SDS, side effects	LKES score significantrly improved in the experimental group. No significant improvement in the other outcomes
Harvey et al. [11]	2018	Randomized Clinical Trial	Patients with CRSwNP and CRSsNP after ESS	44	Interventional group: 51.6 ± 11.9; Placebo group 48.8 ± 14.1	Mometasone irrigation vs mometasone spray	2 mg	240 ml squeeze bottle + spray	Prednisone postoperatively	52	Mometasone irrigation vs mometasone spray	Post-op VAS, SNOT22 13-point Likert score of overall sinonasal function	VAS, SNOT 22 and the global sinonasal function score were all better in the mometasone irrigations group (p < 0.001)
Li et al. [12]	2021	Prospective cohort study	Patients with eosinophilic CRS after ESS and had a postsurgical corticosteroid irrigation regimen	222	54.8 ± 13.6	Budesonide or Bethamethasone	1 mg	240 ml squeeze bottle	Variable	Median duration: 114.4	Pretreatment state	Symptom improvement (questionnaire, SNOT22)	Clinically meaningful change on the 22-item Sinonasal Outcomes Test and the nasal subdomain score was maintained at the last follow-up in 134 patients (67.0%)
Thanneru et al. [13]	2020	Randomized Clinical Trial	Patient with allergic CRSwNP after ESS	60	30.5	Budesonide + saline vs saline	4 mg	250 ml (low pressure, high volume)	Not mentioned	10	Comparison between experimental and control group and with preoperative	SNOT- 22, Lund–Kennedy endoscopy (LK) score	Overall benefit on symptom score during Budesonide irrigation use
Kang et al. [14]	2017	Prospective cohort study	Asthma patients who underwent ESS and took repeated topical or systemic steroid over 6 months due to recurrence or worsening of the disease after ESS	12	49.9 ± 0.9	Budesonide + saline	0.5 mg/2 ml 2/die	250 ml squeeze bottle	Yes	4.8 ± 0.9 months (theorical 24 weeks)	Pretreatment state	SNOT-22 Secondary outcome: Lund-Kennedy endoscopy (LK) score Total amount of oral steroids and inhaled steroids used	Nasal irrigation with budesonide is an effective postoperative treatment for CRS with asthma, which recurs frequently, reducing the oral steroid intake. SNOT-22 and LK score improved
Luz-Matsumoto et al. [15]	2021	Retrospective observational study	Pateints with CRS treated form 3 to 6 month with cortivosteroid nasal spray or corticosteroid nasal irrigations	257	55.5 ± 13.2	Irrigations with: budesonide drops or bethametasone cream	Irrigations: 0.5 mg bethamethasone cream; 1 mg or 0.5 mg of budesonide; spray: 400 ug	250 ml squeeze bottle	None	between January 2013 and December 2019	Pretreatment state	SNOT22 and Lund-Kennedy Endoscopic Score (LKES) pre and post treatment	Both CSNI and CSNS promoted improvement in the nasal endoscopy, with high rates of subjective improvement and low rates of adverse events and exacerbations in the overall sample and in the CRSwNP sample. Among the CSNI therapies, 1% compounded budesonide drops showed better results than betamethasone cream, with the 1000 ug dose showing better results than the 500 ug dose
Ishak et al. [16]	2021	Randomized Clinical Trial	Allegic CRS (no previous ESS)	99	39.17 ± 17.23	Budesonide + saline vs saline	0,6 mg	250 ml squeeze bottle	Not mentioned	24	Comparison between experimental and control group and with preoperative	SNOT-22, endoscopic nasal examination (turbinate hypertrophy, mucosal oedema and secretions), eosinophil count	Patients treated with budesonide had significant improvement in total SNOT-22 score and in the endoscopic findings No significant improvement of blood eosinophil count in patients treated with either budesonide or saline nasal irrigation
Brown et al. [17]	2021	Prospective cohort study	Patients with CRSwNP and CRSsNP after ESS	14	52,2 ± 14,5	Mometasone	4 mg	240 ml irrigations	None	12	Pretreatment state	Impact on serum cortisol SNOT-22	Mometasone irrigations did not cause HPA axis suppression in patients with refractory CRS post-FESS with normal baseline cortisol levels SNOT-22 improved
Rawal et al. [18]	2012	Randomized Clinical Trial	Patients with CRSwNP after ESS	50	46.5	Budesonide	1 mg	240 ml squeeze bottle	None	24	Comparison between experimental and control group and with preoperative	SNOT22, Rhinosinusitis outcomes measurement test (RSOM-31), Rhinosinusitis Disability Index (RSDI), Phenyl Ethyl Alcohol (PEA) threshold test and University of Pennsylvania Smell Identification Test (UPSIT) between the two groups pre and post treatment	Disease specific quality of life is improved with both normal saline irrigations and normal saline plus budesonide irrigations. There is no significant difference in the degree of improvement in the short or long term period between the two. Sense of smell is not improved by any of the treatments
Kosugi et al. [19]	2015	Prospective uncontrolled intervention trial	Patients with CRSwNP and CRSsNP after ESS	16	50.9 ± 10.3	Budesonide	1 mg	20 ml siringe	None	12	Pretreatment state	SNOT22 and Lund-Kennedy Endoscopic Score (LKES) pre and post treatment	Significant improvement of SNOT-22and Lund-Kennedy scores
Jiramongkolchai et al. [20]	2020	Randomized Clinical Trial	Patients with CRSsNP who never underwent ESS. Nasal saline irrigation + mometasone nasal spray vs mometasone nasal irrigation + saline nasal spray	53	48	Mometasone	100 ug per nostril (spray); 2.4 mg (irrigation)	240 ml squeeze bottle and nasal spray	None	16	Comparison between the two arms	SNOT22 pre and post treatment between the two arms	The addition of mometasone to the nasal irrigation was associated with a greater improvement in SNOT-22 score when compared with mometasone nasal spray

**Table 2:** Steroid high-volume irrigations in allergic rhinitis–efficacy

Study	Year	Study type	Patient selection criteria	Participant number	Mean age (years)	Type of steroid	Daily dose	Delivery method	Concomitant systemic steroid use	Duration of the study (weeks)	Comparison	Primary endpoint	Main results
Periasamy et al. [21]	2020	Randomized Clinical Trial	Allergic Rhinitis (AR)	52	Group A: 36.07; group B 31.42	Budesonide hypertonic saline nasal irrigation + antihistamine vs Hypertonic saline nasal irrigation + antihistamine	0.5 mg/2 ml	120 ml squeeze bottle	None	4	group A vs group B and pretreatment state	Intraparticipant change of SNOT22 compared between Group A and Group B	There was a statistically significant improvement in SNOT-22 scores in group A when compared to group B (P = .012)
Ishak et al. [16]	2021	Randomized Clinical Trial	AR (no previous ESS)	99	39.17 ± 17.23	Budesonide + saline vs saline	0,6 mg	250 ml squeeze bottle	Not mentioned	24	Comparison between experimental and control group and with preoperative	SNOT-22, endoscopic nasal examination (turbinate hypertrophy, mucosal oedema and secretions), eosinophil count	Patients treated with budesonide had significant improvement in total SNOT-22 score and in the endoscopic findings No significant improvement of blood eosinophil count in patients treated with either budesonide or saline nasal irrigation

**Table 3:** Steroid high-volume irrigations in chronic rhino sinusitis–safety

Study	Year	Study type	Patient selection criteria	Participant number	Mean age (years)	Type of steroid	Daily dose	Delivery method	Concomitant systemic steroid use	Duration of the study (weeks)	Comparison	Safety endpoint	HPA axis suppression	IOP increase	Other side effects
Zi‐zhen Huang et al. [10]	2018	Case series (prospective)	Patients with CRSwNP and CRSsNP after ESS	60	Experimental group: 37.15 ± 8.623; Control group 38.10 ± 15.579	Budesonide + saline vs saline alone	Not reported	Not reported	Variable	12	Comparison between experimental and control group	side effects (nasal burning sensation, nasal itching, nasal pain, epistaxis, headache, ear pain, cough, nausea and vomiting, postnasal drip, aural fullness and dizziness)	–	–	No impact on side effect
Smith et al. [22]	2015	Case series (retrospective)	Patients with CRSwNP and CRSsNP after ESS	35	49.5 (20–77)	Budesonide + saline	2 mg	Not reported	Inhaled corticosteroids 18 pt (62%)	38.2 months (15–96)	Pretreatment state	AM serum cortisol levels, cosyntropin stimulation test	No	–	–
Seiberling et al. [23]	2013	Prospective study	Group 1: patients already on budesonide irrigations for at least 4 weeks, Group 2: budesonide irrigations for 4 weeks	18	57.2	Budesonide	1 mg	240 ml squeeze bottle	None	4	Pretreatment state	Intraocular pressure (IOP) pre and post treatment	–	No	–
Man et al. [24]	2013	Prospective cohort study	Patients with CRSwNP and CRSsNP after ESS	32 (23 compled, 9 withdrew)	53.3 ± 13.7	Fluticasone propionate + saline	6 mg	240 ml (low pressure, high volume)	None	6	Pretreatment state	Salivary cortisol, intraocular pressure, and the presence of posterior subcapsular cataracts	No	No	No progression of cataract
Dawson et al. [25]	2017	Prospective cohort study	CRS with previous ESS	30	53.9 ± 15.6	Betamethasone	1 ml of 0.5 mg/g	Squeeze bottle	None	6	Pretreatment state	Impact on serum and 24-h urinary free cortisol	No (decrease 24-h urinary free cortisol, clinically not significant)	–	–
Brown et al. [17]	2021	Prospective cohort study	Patients with CRSwNP and CRSsNP after ESS	14	52,2 ± 14,5	Mometasone	4 mg	240 ml irrigations	None	12	Pretreatment state	Impact on serum cortisol	No	–	–
Soudry et al. [26]	2015	Retrospective case-series	All patients who received nasal irrigation with budesonide 0.5 mg for at least 6 month	48	54.5	Budesonide	0.5 mg	240 ml squeeze bottle	None	156	Evaluation of hypothalamic–pituitary–adrenal axis (HPAA) function and IOP	Cosyntropin test results and IOP	Subclinical (23%); associated with concomitant use of nasal steroid sprays and/or pulmonary steroid inhalers	None	–

**Table 4:** Risk of bias assessment

Study	Type of study	RoB
Tait et al	Randomized Clinical Trial (RCT)	Some concerns
Kosugi et al	RCT	High
Harvey et al	RCT	Low
Rawal et al	RCT	High
Jiramongkolchai et al	RCT	Low
Periasamy et al	RCT	High
Huang et al	RCT	High
Ishak et al	RCT	Some concerns
Li et al	Prospective cohort study (PCS)	High
Snidvongs et al	PCS	High
Jang et al	PCS	Low
Luz-Matsumoto et al	Retrospective cohort study (RCS)	High
Papagiannopulos et al	RCS	Some concerns
Talat et al	Case-series	Some concerns
Kang et al	Case-series	Some concerns
Maheshbabu et al	Prospective case–control study	Some concerns
Dawson et al	Non-randomized Clinical Trial	Some concerns

## Results

The flow diagram in Fig. 1 summarizes the selection process. After the screening of titles and abstracts, the investigators achieved a consensus to include 38 studies for full-text assessment. Of those 18 studies were excluded because they did not fit the study’s purpose yielding the inclusion of 20 papers. Selected papers were divided as follows: 13 papers studying the effectiveness of high-volume nasal irrigations with steroids, 4 papers studying their safety and adverse effects 3 papers that evaluated both topics. No studies on cost-effectiveness were found.

The final article selection comprehends 8 Randomized Clinical Trials, 8 prospective cohort trials or case-series, 4 retrospective observational studies. Regarding the type of corticosteroid diluted in the nasal irrigations in 15 studies budesonide was used, while mometasone, betamethasone and fluticasone propionate were used in 3, 4 and 1 studies respectively. The most employed method to perform nasal irrigations was the 240 ml squeeze-bottles (14/20 studies). The detailed characteristics of the selected studies are shown in Tables 1–3.

Regarding the risk of Bias of the randomized clinical trials 2 studies were classified as having low risk, 3 having “some concerns” and 3 having “high risk”. Considering the prospective cohort studies or case-series 6 had high risk, while the other 2 presented low risk of bias. The 4 retrospective observational studies had a high risk of bias. The results of the RoB assessment are summarized in Table 4.

## Corticosteroid high volume irrigations vs other topical delivery methods

We found one double-blind, placebo-controlled trial of 44 patients assessing the use of large-volume steroid irrigations vs nasal corticosteroid spray after sinus surgery11. Each patient used a nasal spray and large-volume irrigation and was blinded to which device contained the steroid. The dose of 2 mg of mometasone was equal for both the nasal spray and the high-volume irrigations. The groups were evaluated at 12 months and, although both groups improved their nasal symptoms, it was the corticosteroid irrigation group that showed greater improvement in nasal blockage (− 69.91 ± 29.37 vs − 36.12 ± 42.94; p = 0.029) and modified Lund-Kennedy scores (7.33 ± 11.55 vs 21.78 ± 23.37; p = 0.018). Moreover at 12 months the symptoms in the nasal spray group had begun to worsen and the overall 12 month symptom visual analog score was better in the high-volume irrigation group. In addition, the study from Jiramongkolchai et al. analysed the usage of mometasone irrigations vs nasal sprays in the context of CRSsNP in 43 patients who never underwent FESS20. Patients were randomized to receive 8 weeks of either Mometasone furoate spray or mometasone nasal irrigations. The mometasone lavage group had a clinically meaningful improvement in SNOT-22 scores with a proportion difference of 17% (95% confidence interval [CI], − 9% to 44%). The least-squares (LS) mean difference between the 2 groups for SNOT-22 was − 8.6 (95% CI, − 17.7 to 0.58; p = 0.07), whereas the LS mean difference between the 2 groups for Lund-Kennedy endoscopy scores was 0.16 (95% CI, − 0.84 to 1.15; p = 0.75).

Luz-Matsumoto et al. investigated in a retrospective observational study the clinical response of nasal irrigation with 1% compounded budesonide drops or betamethasone cream compared to nasal sprays utilized in patients with CRS15. Corticosteroid nasal irrigations and nasal sprays improved the Lund-Kennedy endoscopic score without any statistical significance between the two treatments. However more adverse events were recorded in the corticosteroid nasal irrigation group, especially in the group using bethametasone cream [15]. Patients experienced ear fullness, epistaxis, nasal irritation, epigastric pain and nausea but not all these symptoms can be reasonably attributed to nasal irrigations with steroids and were recorded in a small number of cases [15].

## Corticosteroid high volume irrigations vs saline irrigations

Tait et al. evaluated the incremental effect of adding budesonide to large-volume, low-pressure saline sinus irrigation in a double-blind, placebo-controlled, randomized clinical trial [9]. All the 61 participants with CRS received a sinus rinse kit containing either budesonide (treatment group) or lactose (control group) capsules and were instructed to use the solution once daily for 30 days. The average change in SNOT-22 scores was 20.7 points for those in the budesonide group and 13.6 points for those in the control group, for a mean difference of 7 points in favor of the budesonide group (95% CI, − 2 to 16). A total of 23 participants (79%) in the budesonide group experienced a clinically meaningful reduction in their SNOT-22 scores compared with 19 (59%) in the control group, for a difference of 20% (95% CI, − 2.5% to 42.5%). The average change in endoscopic scores was 3.4 points for the budesonide group and 2.7 points for the control group. Another study in the postoperative setting of CRSwNP was conducted by Rawal et al. where 50 patients were prospectively enrolled to normal saline or normal saline + budesonide arms18. Patients were evaluated at pre-operative and three post-operative visits (POV): POV1 (1–2 weeks post-op), POV2 (3–8 weeks post-op), and POV3 (3–6 months post-op). By POV2 and POV3, patients experienced a significant improvement in all three QOL surveys, although the degree of improvement between arms was not significant up through POV3. Neither arm experienced significant olfactory improvement up through POV3. There was no statistically significant difference between the two groups.

A study by Thanneru et al. evaluated the efficacy of budesonide nasal irrigations in the post operatory management of chronic allergic rhinosinusitis with polyps [13]. A total of 60 patients was divided in 2 groups. One group of patients received budesonide nasal douching in addition to regular care. Both groups were evaluated endoscopically at 1, 2, 6 and 10 weeks after surgery. The average postoperative SNOT22 was 29.4 in patients who used the standard postoperative regimen and 15.8 in patients who had budesonide added to their douching solutions. The average endoscopy score was 2.2 for patients who did receive budesonide as compared to 2.9 for patients who did not receive budesonide nasal douching.

The use of budesonide nasal irrigations was studied also in AR by Ishak et al. with a randomised controlled study involving 99 patients diagnosed with AR, half of whom were treated with saline nasal irrigation and the other half with budesonide and saline nasal irrigation [16]. Patients treated with budesonide nasal irrigation had significant improvement in total SNOT-22 score (mean improvement: 13.93; 95% CI: 8.05, 19.81 P < 0.001) and improvement in the endoscopic nasal examination findings, such as nasal mucosa oedema and secretions (mean improvement: 0.42; 95% CI: 0.25, 0.59, P < 0.001). The group of Periasamy et al. has conducted a single-center, double-blind, RCT in which 52 patients with AR were divide into 2 groups to receive either placebo respule or buffered hypertonic saline nasal irrigation with a budesonide respule [21]. Patients were assessed at baseline and 4 weeks. The budesonide irrigation group was found to have significantly better improvement than the saline nasal irrigation group with the SNOT-22 scores (P = 0.012) and VAS scores (P = 0.007). However, the difference in the clinical response between the 2 groups was not significant (P = 0.268). Kang et al. sought to determine whether the addition of budesonide irrigations decrease the need for systemic corticosteroid therapy [14]. Twelve patients with CRSwNP and asthma under oral corticosteroid treatment were evaluated 6 months after initiation of budesonide irrigations. There was significant improvement with corticosteroid irrigations in SNOT- 22(30.8 ± 14.4vs14.2 ± 8.7; p = 0.03) and LK (7.4 ± 4.7 vs 2.2 ± 2.7; p < 0.001) scores at 6 months [14]. There was also significantly less oral corticosteroid use in the 6 months after initiation of budesonide irrigations (397.8 ± 97.6 mg vs 72.7 ± 99.7 mg; p < 0.001), with 50% of the patients requiring no systemic corticosteroid treatment after the initiation of budesonide irrigations [14]. One study from Kosugi et al. evaluated the benefits of budesonide irrigations in a difficult-to-treat CRS population (absence of appropriate clinical control level despite sinonasal surgery, intranasal corticosteroids and up to two cycles of antibiotics or systemic corticosteroids in the last year) [19]. Patients were given budesonide irrigations at a dosage of 1 mg/day and the SNOT-22 and LK scores were assessed before and after 3 months. The SNOT-22 scores improved significantly after the initiation of budesonide irrigations (50.2 ± 19.3 vs 29.6 ± 20.4; p = 0.006), as did LK scores (8.8 ± 3.3 vs 5.1 ± 4.4; p = 0.01) [19].

## Corticosteroid high volume irrigations vs no irrigations

Jang et al. performed a retrospective review of 60 patients who were being treated with budesonide irrigations (1 mg/d) postoperatively [8]. All these patients served as their own controls during a time they were not compliant with therapy and their 20-item SNOT (SNOT- 20), and LK scores were evaluated [8]. Thirty-three patients (55%) had lower SNOT-20 scores on corticosteroid irrigations. The mean postoperative SNOT-20 scores on budesonide therapy were significantly lower than when not using the irrigation (12.5 ± 10.4 vs 15.1 ± 12.1; p < 0.05); however, the LK scores were not significantly improved (4.7 ± 3.5 vs 5.7 ± 3.7; p = 0.08). When eCRS patients were on budesonide irrigations, they had significantly lower SNOT-20 (11.7 ± 11.6vs15.3 ± 13.0; p = 0.04) and LK (4.4 ± 3.4vs 5.9 ± 3.0; p = 0.02) scores. Whereas patients with Samter’s triad only had significantly lower SNOT-20 scores (14.9 ± 8.4 vs 21 ± 10.3; p = 0.04), and allergic fungal sinusitis showed no significant difference for SNOT-20 (12.3 ± 7.4 vs 8.7 ± 7.2; p = 0.13) or LK (2.9 ± 2.3vs2.5 ± 2.6; p = 0.6) scores.

In the study of Snidvongs et al. the authors prospectively investigated the efficacy of budesonide or betamethasone irrigations 1 mg/day in the postoperative care of CRS2. Baseline and post- treatment symptom scores (2.6 ± 1.1 vs 1.2 ± 1.0), SNOT- 22 scores (2.2 ± 1.1 vs 1.0 ± 0.8), and endoscopy scores (6.7 ± 3.0 vs 2.5 ± 2.0) revealed significant improvement (all, p < 0.001). Patients with high tissue eosinophilia (> 10/high power field [HPF]) had significantly more improvement on symptom score (1.9 ± 1.4 vs 1.1 ± 1.0, p = 0.04), SNOT-22 score (1.6 ± 1.3 vs 1.0 ± 0.8, p = 0.03), and endoscopy score (5.12 ± 3.4 vs 3.06 ± 3.0, p = 0.01) than those without.

A study similar to the previous one was made by Li et al. in which patients with eCRS were given budesonide or betamethasone nasal irrigations 1 mg/day after radical surgery performed with removal of all intersinus bony partitions to create a neo-sinus cavity12. Of the 222 patients at 6 months, 195 (87.8%) had well-controlled disease, 16 (7.2%) had polyp recurrence, 7 (3.2%) continued to receive long-term oral corticosteroid therapy, 5 (2.3%) received biologic therapy, and 8 (3.6%) underwent a revision polypectomy. Clinically meaningful change on SNOT 22 score was maintained at the last follow-up in 134 patients (67.0%).

## Safety

A prospective cohort study by Brown et al. evaluated the possibility of short-term hypothalamic–pituitary–adrenal axis (HPA) suppression with high-volume, high-dose nasal mometasone irrigation (2 mg/240 mL twice a day for 12 weeks) in 14 postsurgical patients with chronic rhinosinusitis, dosing morning cortisol serum levels [17]. Except for one patient, the levels of pre- and post-treatment morning cortisol did not differ significantly and were within the normal range; no evidence of HPA axis suppression was found (P = 0.915). The only patient who had a low post-treatment cortisol level received an intraarticular steroid shot several days prior to the blood draw and this value returned to normal at a late recheck.

Dawson et al. in 2017 published a study on 30 patients, previously treated with FESS for CRS, that performed nasal irrigation with betamethasone cream (0.5 mg/200 mL once a day) for 6 weeks, evaluating both efficacy, through SNOT-22 score, and safety, assessing the impact on pre- and post-intervention serum and 24-h urinary free cortisol25. SNOT-22 scores improved (41.13 ± 21.94 vs 23.4 ± 18.17; p < 0.001), while serum cortisol levels were unchanged (p = 0.28). On the other side, mean 24-h urinary free cortisol levels decreased (47.5 vs 41.5 nmol per 24 h; p = 0.025), but these changes are unlikely to be indicative of HPA suppression and were considered clinically negligible, due to the patients’ persistent absence of symptoms.

Man LX et al. evaluated the effect of intranasal fluticasone propionate irrigations (3 mg/240 mL twice a day for 6 weeks) on salivary cortisol, intraocular pressure (IOP), and posterior subcapsular cataracts in a prospective study on 23 patients who had previously undergone FESS for CRS24. Salivary cortisol levels where normal before and after therapy in all patients and there was no statistical difference in mean salivary cortisol levels pre-treatment and post-treatment (0.294 vs 0.392 μg/dL; p = 0.27). No clinical or statistical difference was found in mean intraocular pressure before or after therapy (13.3 vs 13.3 mmHg; p = 0.86) and also no subjects developed a posterior subcapsular cataract.

The impact of nasal irrigation with steroid on IOP was studied also by Seiberling et al. [23] The authors divided the patients in two groups: the first, retrospective, consisting of 10 patients on intranasal budesonide irrigations (1 mg/240 mL twice a day) for an average duration of 6.3 months at the time of enrollment (range, 1–22 months); the second was composed of 8 patients prospectively enrolled and treated with the same regimen for at least 4 weeks (average length of therapy of 5.89 weeks, range 4–8 weeks). IOP was measured once at the time of enrolment in the first group and before and after at least 4 weeks of therapy in the second one. Only 1 patient of the retrospective group had a single eye pressure above 21 mmHg, while none of the patients in second group had a significant change in IOP (p < 0.05) or IOP over 21 mmHg. Overall, intranasal budesonide irrigations given for a period of at least 1 month do not appear to increase IOP; a longer period, could theoretically elevate IOP. However, considering results from group 1 with an average duration of therapy of 6.3 months, the authors concluded that this is unlikely to happen.

Smith et al. evaluated the long-term safety of these kind of nasal irrigation [22]. In their study, 35 patients with CRS managed with Budesonide nasal irrigation (2 mg/day) were involved and the mean therapy duration at testing was 38.2 months. 62% of patients were taking also concurrent inhaled corticosteroids for asthma. Primary outcome measure was morning serum cortisol levels, which, if greater than 500 nmol/L can exclude HPA axis suppression in these patients. The mean ± standard deviation morning serum cortisol was 431.2 ± 146.9 nmol/L (normal = 200–650 nmol/L). In patient with value less than 500 nmol/L, subsequent cosyntropin stimulation tests demonstrated no evidence of HPA axis suppression in any case.

In a similar study by Soundry et al., a total of 48 patients were assessed, with a mean duration of Budesonide irrigations (0.5 mg/240 once or twice a day) of 22 months26. Eleven patients (23%) had abnormally low stimulated cortisol levels, but none of them experienced symptoms of adrenal suppression. Three of 4 patients who repeated the study being off budesonide for at least 1 month returned to near normal levels and, anyway, all these patients were able to continue budesonide irrigations under the supervision of an endocrinologist without clinical manifestations of adrenal insufficiency. Concomitant use of both nasal steroid sprays and pulmonary steroid inhalers was significantly higher (p = 0.021) in the group of patients with low stimulated cortisol levels. IOP was also tested in 46 patients and was found to be within normal limits in all cases.

Huang et al. published a randomized controlled study on steroid irrigation in 60 CRS patients after undergoing ESS, half of whom were treated with saline nasal irrigation and the other half with budesonide nasal irrigation for 12 weeks [10]. All patients were assessed before ESS and after the therapy via the Lund–Kennedy endoscopic score (LKES), the symptom visual analog scale (VAS), the 22-item Sino-Nasal Outcome Test (SNOT-22), the Short-Form 36-Item Questionnaire (SF-36), the Self-Rating Anxiety Scale (SAS), the Self-Rating Depression Scale (SDS) and a side effects scale. LKES were significantly better in the experimental group than in the control group at 3 months after ESS (p = 0.009), while no significant improvement in the other outcomes. Side effects reported (nasal burning sensation, nasal itching, nasal pain, epistaxis, headache, ear pain, cough, nausea and vomiting, postnasal drip, aural fullness and dizziness) were not significantly different in the 2 groups.

## Discussion

### Effectiveness

Chronic rhinosinusitis (CRS) and allergic rhinitis (AR) are among the most common chronic respiratory diseases affecting millions of people worldwide. In Europe, it is estimated that about 11% of adults are affected by CRS [27]. In Italy, solid epidemiological data are lacking, although some estimates the prevalence of acute and chronic RS to be around 20% [28]. The economic burden of these diseases is also substantial, with indirect costs that exceed direct ones and can reach as much as 2 billion of euros/year, as calculated for Netherlands in the study by Lourijsen et al. [29].

The main goal of surgery in CRS is to create a more functional anatomy and allow for better outflow of mucous secretions and greater delivery of topical therapy [1]. Prior research has demonstrated that when sinuses are extensively opened, especially for frontal and sphenoid ones, topical medication can penetrate more effectively than in cases where the sinuses are only partially opened or have not undergone surgical intervention [3].

Reviewed studies reported delivery devices with volumes ranging from 90 to 250 mL. Delivery of at least 100 mL has been shown to reliably penetrate the paranasal sinuses after surgery [30]. Only in the retrospective case series by Jang et al., patients were instructed to irrigate each nasal fosse with 45 mL of a solution containing 0.5 mg of Budesonide, still achieving an improvement on symptoms scores [8]. On the other hand, in the study of Zi-Zhen Huang et al., daily dose of Budesonide and volume and type of delivery method were not reported and no significant improvement on symptoms scores was found; one of the factors influencing this result might be too low irrigation volume [10]. Eventually, all the reviewed studies in which irrigation volume was adequate (> 100 mL) showed significant improvement in symptoms control [Tables 1, 2]. In the study by Harvey et al., high-volume corticosteroid irrigations were compared to nasal corticosteroid sprays and, despite both groups of patients receiving identical doses of mometasone (2 mg/d), improvement was observed in the high-volume irrigation group, as a result of a more effective delivery of topical medication to the sinus cavity [11].

Several corticosteroids were employed in reviewed studies: Betamethasone in 4, Fluticasone in 1, Mometasone in 3, and Budesonide in 15 studies [Tables 1, 2]. Effectiveness of treatment was not studied for Fluticasone, that were instead examined only for their safety [24]. However, in the other studies, the type of corticosteroid used did not produce any significant differences in outcomes. Thus, the key to achieving efficacy lies in selecting a corticosteroid preparation that can easily dissolve in a saline irrigation and delivering through high-volume devices to the paranasal sinuses, rather than the specific type of corticosteroid utilized [15].

Three studies did not find any significant benefit when using corticosteroid irrigations. In Tait’s study, there was a slight improvement, but it was not statistically significant9. This could be due to the short duration of treatment (4 weeks) or the medication not being able to reach the sinuses since most patients had not undergone surgery. In the study of Zi-Zeng Huang, patients were previously operated and significant improvement in endoscopic score was found, however this did not translate in improvement of the nasal symptoms [10]. As mentioned above, delivery method and volume were not reported. Rawal’s study also did not show any benefit of nasal Budesonide irrigation compared to saline ones; however, it is difficult to establish the specific efficacy of the topic therapy since patients were treated with systemic corticosteroid as well in the postoperative period and they received the final evaluation within only 3 to 6 months from treatment initiation [18], while most authors advocate that outcomes should be measured at least 6–12 months after surgery [3]. However, all other studies evaluating the efficacy of corticosteroid irrigations have shown an overall benefit, regardless of the dose, corticosteroid used, timing of follow-up, etiology of disease, or surgical state, when compared to saline irrigations, nasal corticosteroid sprays, or no topical treatment [Table 1].

## Safety

With the increasing use of topical corticosteroid irrigations, concerns about their long-term safety persist. Systemic corticosteroids have various risks associated with prolonged use, and any treatment replacing them should balance efficacy with the patient’s safety. Reviewed studies have shown that the use of topical corticosteroid irrigations for up to 39 months has not resulted in adrenal suppression or significant changes in intraocular pressure, indicating their safety for long-term use [Table 3]. Indeed, despite the higher quantity, less than 5% of the total rinse remains in the nose ensuring a final available dose equivalent to that used for nasal sprays [31]. However, in the study of Soudry et al. a subclinical suppression of the hypothalamic–pituitary- adrenal axis was found to be associated to the concomitant use of nasal steroid sprays or pulmonary steroid inhalers [26]. Moreover, we should remain vigilant about the potential risk of central serous choreoretinopathy (CSC) associated with corticosteroid use. CSC is the fourth most common retinal disease and has been linked to corticosteroid use, with a higher odds ratio observed in inhalant/nasal corticosteroid use than oral corticosteroid use [32]. In particular, patients who already receive a systemic corticosteroid therapy for a comorbid condition such as asthma might be more susceptible to its adverse effects. On the other hand, we must consider that the increased systemic absorption with the usage of corticosteroid nasal rinses is often associated with a reduced necessity for oral or inhalatory administration for patients with comorbid asthma due to better overall airway inflammation control [14]. Although off-label topical corticosteroid irrigations have proven to be safe, it is worth highlighting that clinicians still bear the responsibility to monitor patients and prevent adverse events by taking into consideration not only the effects of nasal steroid therapies but also the overall steroid load associated with topical or systemic treatment of frequent comorbid conditions (e.g. asthma, atopic dermatitis).

The evidence collected is extremely variable and presents various confounding factors such as: previous surgery and its extension, CRS endotypes and characteristics (CRSwNP and CRSsNP), type of corticosteroid used during rinses and its dosage, capacity and types of the delivery devices. Nevertheless, the safey profile of HVSC is confirmed by all the studies in the present review, whereas, in the few studies in which HVSC was compared directly to the use of corticosteroid sprays (i.e., mometasone), their efficacy seemed to be even higher [11, 20].

Recent research on the commercial corticosteroid Pulmicort revealed significant antibacterial effects on both planktonic and biofilm forms of Staphylococcus aureus and methicillin-resistant Staphylococcus aureus [33]. This effect, observed in the presence of budesonide and its excipients, particularly ethylene diamine-tetra acetic acid (EDTA), suggests an additional dimension to the therapeutic impact of corticosteroid irrigations. Notably, EDTA demonstrated antibacterial properties even at low concentrations used in topical preparations. This finding raises intriguing possibilities regarding the role of corticosteroids in addressing bacterial aspects of chronic rhinosinusitis.

Despite evidence, HVSC is still poorly adopted, and patients are often referred to expensive treatments, like revision surgery or biologic therapy, rather than trying it in advance. Implementing HVSC could be an effective strategy not only for highly symptomatic patients, but, with careful selection of those who could control symptoms even with this therapy alone, also to optimize cost–benefit in the management of CRSwNP. Literature regarding cost-effectiveness of HVSC, especially compared to surgical or biologic therapies, is lacking and this aspect may be important for future directions on the topic.

## Conclusion

HVSC represent a safe and effective alternative to topical nasal sprays formulations regarding pre and postoperative care of chronic rhinosinusitis both with and without nasal polyps. Nevertheless, the exact timing of therapy and the type of steroid that guarantees the maximal efficacy must be better delineated in further studies. Eventually, even if the safety profile of these medications is high, particular attention in HPAA suppression must be taken especially in subject under inhalatory/systemic corticosteroid therapy for other comorbidities.
